# Digital Behavior Change Intervention Designs for Habit Formation: Systematic Review

**DOI:** 10.2196/54375

**Published:** 2024-05-24

**Authors:** Yujie Zhu, Yonghao Long, Hailiang Wang, Kun Pyo Lee, Lie Zhang, Stephen Jia Wang

**Affiliations:** 1 School of Design, The Hong Kong Polytechnic University Hong Kong China (Hong Kong); 2 Laboratory for Artificial Intelligence in Design Hong Kong Science Park Hong Kong China (Hong Kong); 3 Academy of Arts & Design, Tsinghua University Beijing China

**Keywords:** habit formation, digital health, digital behavior change interventions design, behavior change techniques, physical activity, mobile phone

## Abstract

**Background:**

With the development of emerging technologies, digital behavior change interventions (DBCIs) help to maintain regular physical activity in daily life.

**Objective:**

To comprehensively understand the design implementations of habit formation techniques in current DBCIs, a systematic review was conducted to investigate the implementations of behavior change techniques, types of habit formation techniques, and design strategies in current DBCIs.

**Methods:**

The process of this review followed the PRISMA (Preferred Reporting Item for Systematic Reviews and Meta-Analyses) guidelines. A total of 4 databases were systematically searched from 2012 to 2022, which included Web of Science, Scopus, ACM Digital Library, and PubMed. The inclusion criteria encompassed studies that used digital tools for physical activity, examined behavior change intervention techniques, and were written in English.

**Results:**

A total of 41 identiﬁed research articles were included in this review. The results show that the most applied behavior change techniques were the self-monitoring of behavior, goal setting, and prompts and cues. Moreover, habit formation techniques were identified and developed based on intentions, cues, and positive reinforcement. Commonly used methods included automatic monitoring, descriptive feedback, general guidelines, self-set goals, time-based cues, and virtual rewards.

**Conclusions:**

A total of 32 commonly design strategies of habit formation techniques were summarized and mapped to the proposed conceptual framework, which was categorized into target-mediated (generalization and personalization) and technology-mediated interactions (explicitness and implicitness). Most of the existing studies use the explicit interaction, aligning with the personalized habit formation techniques in the design strategies of DBCIs. However, implicit interaction design strategies are lacking in the reviewed studies. The proposed conceptual framework and potential solutions can serve as guidelines for designing strategies aimed at habit formation within DBCIs.

## Introduction

### Background

Physical activity has been proved to play an essential role in lifestyle management [1]. Incorporating regular physical activity into daily routines to form habitual healthy behavior is an effective strategy for reducing risks of chronic diseases (eg, hypertension, stroke, or diabetes) and maintaining a healthy lifestyle [1]. Common types of physical activities in daily life include walking, bicycling, hiking, swimming, and participating in various sports. According to the guidelines of the World Health Organization and the American College of Cardiology [1,2], adopting 90 to 150 minutes of aerobic exercise during a week is recommended. However, >1.4 billion adults do not get sufficient physical activity, particularly in high-income countries [3].

Digital behavior change interventions (DBCIs) provide potential solutions to increase physical activity levels and maintain healthy lifestyle habits. With the development of ubiquitous computing and Internet of Things (IoT), smartphones are gradually becoming the center of mobile devices. Research on mobile health or eHealth has developed rapidly after 2010, whereas mobile apps are a commonly used medium in DBCIs [4]. Various sensors are embedded in smartphones or wearable devices, which have the ability to track a large amount of personal data and perceive changes in surrounding environments, such as routines, preferences of individuals, and context information [5-7]. Compared with traditional interventions, the real-time health status of patients could be captured and analyzed in real time because of the ability of big data [6,8]. Meanwhile, integrating artificial intelligence (AI) into health systems could benefit from predicting, identifying, and providing personalized diagnoses [9]. Therefore, research on technology-mediated behavior change interventions has gradually evolved from providing one-size-fits-all solutions to patient-centered approaches, which meet personalized requirements.

Despite the significant progress in DBCIs, prior studies have shown the challenges in maintaining long-term adoption [10,11]. On the one hand, although DBCI solutions have widely applied automatic tracking strategies, the interpretation and feedback of tracking data do not satisfy users’ expectations [10,12,13]. Users may stop adopting DBCI solutions because they perceive uselessness. On the other hand, most DBCI solutions depend on users’ initiative and consciousness to drive goals, which requires users’ effort and frequent proactive interaction with devices [11,14]. However, changes in daily routine may result in additional workflow for tracking. The ability of mobile phones and smart wearable devices to implicitly sense surroundings to infer users’ intentions remains underused. Users may discontinue because of the loss of motivation for long-term tracking.

### Prior Studies of DBCIs for Habit Formation

To engage in long-term tracking of physical activity, habit formation is argued to play an essential role in the health behavior change domain [15,16]. Some research emphasized that habits develop from nonconscious and implicit associations between context and behavior [15,17]. In addition, behavior is not only facilitated by habits but also driven by consciousness [16,18], which corresponds with the dual process theory [19] that behavior results from both conscious and unconscious. Prior studies proposed the theoretical process of habit formation [15,16,20]. Three key elements were summarized to form habits: implementation intention, positive reinforcement, and cues [16,20,21]. Behavior initially acts on consciousness in reflective processes, which drive intention through goals and planning. Regarding maintaining long-term motivation to form habits, cues serve as triggers that are repeated to form associative memories and habitual execution. Positive reinforcements refer to internal and external rewards that are used to accelerate the process of cue-behavior association.

Regarding the integration of theoretical models and design practices in DBCIs, studies from human-computer interaction (HCI) focused on using psychological theories and capabilities of technology to design solutions for human behavior change. For instance, Pinder et al [20] proposed the Habit Alteration Model and a set of design guidelines to illustrate how reflective and automatic processes generate and influence habitual behavior. Meanwhile, prior studies have adopted various behavior change techniques (BCTs) in DBCIs for physical activity. BCTs refer to a series of replicable interventions with the goal of changing behavior [22], which have been widely adopted in DBCIs. Duff et al [23] conducted a systematic review to access the BCTs’ application for chronic disease interventions, which demonstrated that the description of the BCTs’ application was vague. Studies either fail to mention what type of BCTs were used in DBCIs’ design process or link the BCTs types to the functionality of DBCIs. Nyman et al [24] summarized that goal setting, social support, and credible source have potential effects for improving physical activity levels among patients with dementia, although there is no evidence of the effectiveness of BCTs for long-term behavior change. In addition, Direito et al [25] argued that existing DBCIs had minor effects on physical activity for adults through a meta-analysis, which was potentially caused by some commonly applied BCTs only promoting behavior change in the short term instead of behavior maintenance. However, despite prior studies that have demonstrated the effects of DBCIs on behavior change in a short time, studies from the HCI domain emphasized that most existing DBCI solutions still rely on users’ intention and proactive interaction with digital devices [20,26]. On the one hand, Adams et al [26] found that 94% of published DBCIs focused on goal-directed behaviors that aim to promote reflective thinking and motivate behavior change intention, which demands users’ extra attention and effort to engage with technologies. Therefore, the unconscious behavior of users is induced by the subtle stimulus of the surrounding environment, and the automated process is ignored. On the other hand, limited studies have investigated how to design DBCI solutions using characteristics of these technologies, such as context awareness and inferring personal requirements [20,27]. According to a study analyzing apps from the Apple App Store, it has been found that the most common design patterns of DBCIs are setting reminders and determining goals of activity [28], which ignores the characteristics of these technologies in implicitly sensing the users’ intention.

### Objectives

To enhance understanding of the potential of technology and explore future possibilities in designing DBCIs to facilitate long-term adoption, this study aims to investigate the design strategies of habit formation in existing DBCIs for physical activity from the perspective of designing DBCI solutions. To tackle these objectives, a systematic review was conducted to summarize existing studies based on 2 main research questions (RQs):

- RQ1: What types of techniques are applied in DBCIs to facilitate habit formation?
- RQ2: What design strategies leveraging the capabilities and features of digital devices have been investigated in DBCIs for habit formation?

In this paper, habit refers to consistent and long-term performance of healthy behaviors in daily life. Habit formation techniques encompass design strategies and functional implementation solutions that use BCTs and characteristics of technology. As a result, this study contributes in the following ways:

1. Summarizing 32 commonly used habit formation techniques in existing DBCIs.
2. Proposing a conceptual framework that formulates these habit formation techniques from the perspectives of target-mediated and technology-mediated interactions.
3. Identifying the challenges faced by designing DBCIs in the habit formation process and suggesting potential solutions.

## Methods

### Overview

To investigate the limitations of existing studies and opportunities of DBCIs for long-term habit formation, specifically implementations of BCTs, a systematic review was conducted that followed the PRISMA (Preferred Reporting Item for Systematic Reviews and Meta-Analyses) guidelines [29].

### Search Strategy

Web of Science, Scopus, ACM Digital Library, and PubMed were selected and searched for relevant studies published from 2012 to 2022. The search strategy combined concepts for health behavior type (physical activity), technology implementation, and behavior change to form habits. Hence, the following search terms were used: (physical activit* OR step* OR fitness OR exercise) AND (mobile* OR smart* OR wearable* OR digital* OR IoT OR internet of things) AND (behavior change OR habit* OR form habit OR formation of habits).

### Selection Criteria

Articles were selected if they met the following inclusion criteria: (1) the publication is focused on behavior change interventions or habit formation for physical activity, (2) the publication should use digital tools (eg, smartphones, wearables, and IoT devices) for assisting behavior change, (3) the publication should include the entire process of study design and validation results, (4) the aim of the publication should be to use DBCI for personal health management, and (5) the publication is journal articles or conference papers in English. Studies were excluded if they were reviews, magazines, lecture notes, abstracts, books, and other situations that did not meet the inclusion criteria.

### Data Extraction and Analysis

All initial search results were scanned with title and abstract by the first author and 2 research assistants, followed by a group meeting to discuss the disagreements. To gain a comprehensive understanding of the current state of DBCI for habit formation, the fourth, fifth, and sixth authors discussed and determined the following information to be extracted from selected articles:

- BCTs: adopted BCTs were summarized based on selected articles.
- Habit formation techniques: information was concluded based on 3 elements for forming habits, which were goal-directed behavior, positive reinforcement, and cues.
- Validation: information about the objectives of the selected articles was extracted.
- Outcome-related information: outcome measures and study duration were extracted.

The coding of the extracted information was conducted independently by the first 2 authors by using thematic analysis [30]. Subsequently, a group meeting involving the first 2 authors and the third author was held to discuss and review the extracted information. Any disagreements of coding were discussed within the group, and final decisions were made by the third author. The final coding was categorized and summarized into descriptive themes of DBCIs’ implementations, which were reviewed by the senior researchers.

## Results

### Search Results

**Figure 1:**
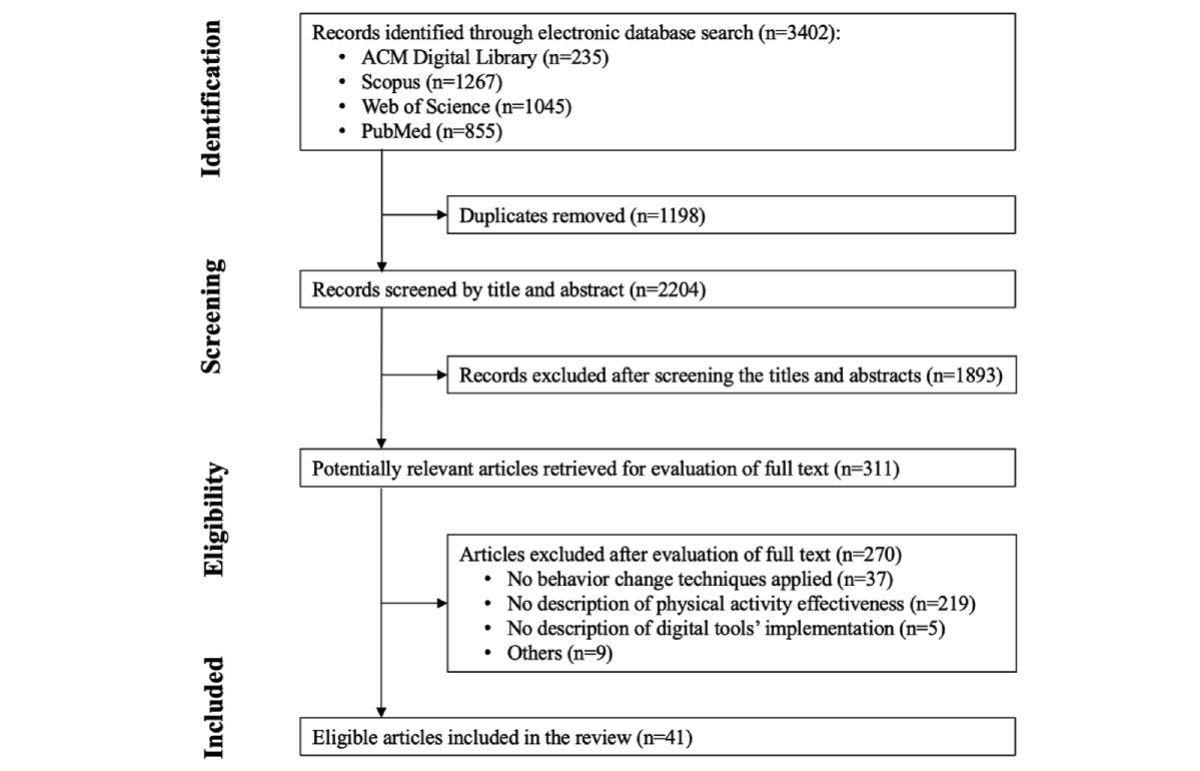
PRISMA (Preferred Reporting Item for Systematic Reviews and Meta-Analyses) flow diagram of the study selection process.

Figure 1 shows the entire selection process. A total of 3402 articles were identified from databases, of which 1198 (35.21%) duplicates were removed, leaving 2204 (64.79%) studies for title and abstract screening. Subsequently, of these 2204 studies, 311 (14.11%) were reviewed in full text, of which 270 (86.8%) were excluded with reasons, and 41 (13.2%) eligible publications were included.

### Frequency for Adopting BCTs

The most frequently applied BCT group used in the selected studies was feedback and monitoring (36/41, 88%), whereas the second and third most applied groups were associations (33/41, 80%) and goals and planning (30/41, 73%). This was followed by social support (27/41, 65%), reward and threat (27/41, 65%), comparison of behavior (22/41, 53%), natural consequences (11/41, 27%), repetition and substitution (11/41, 27%), and shaping knowledge (10/41, 24%). The 5 BCT groups that were less adopted in the selected studies were identity, comparison of outcomes, antecedents, scheduled consequences, and self-belief, with 27% (11/41) of the articles.

The frequency of adopting BCTs across the 41 studies was summarized. Three techniques were adopted in >60% (25/41) of the studies: prompts and cues (33/41, 80%), goal setting (27/41, 65%), and self-monitoring of behavior (25/41, 60%). In addition, 5 BCTs were applied in >30% (13/41) of the studies: feedback on behavior (23/41, 56%), social support (unspecified; 21/41, 51%), social comparisons (18/41, 43%), review outcome goals (16/41, 39%), and nonspecific rewards (16/41, 39%).

### Validation of the Existing Studies

**Figure 2:**
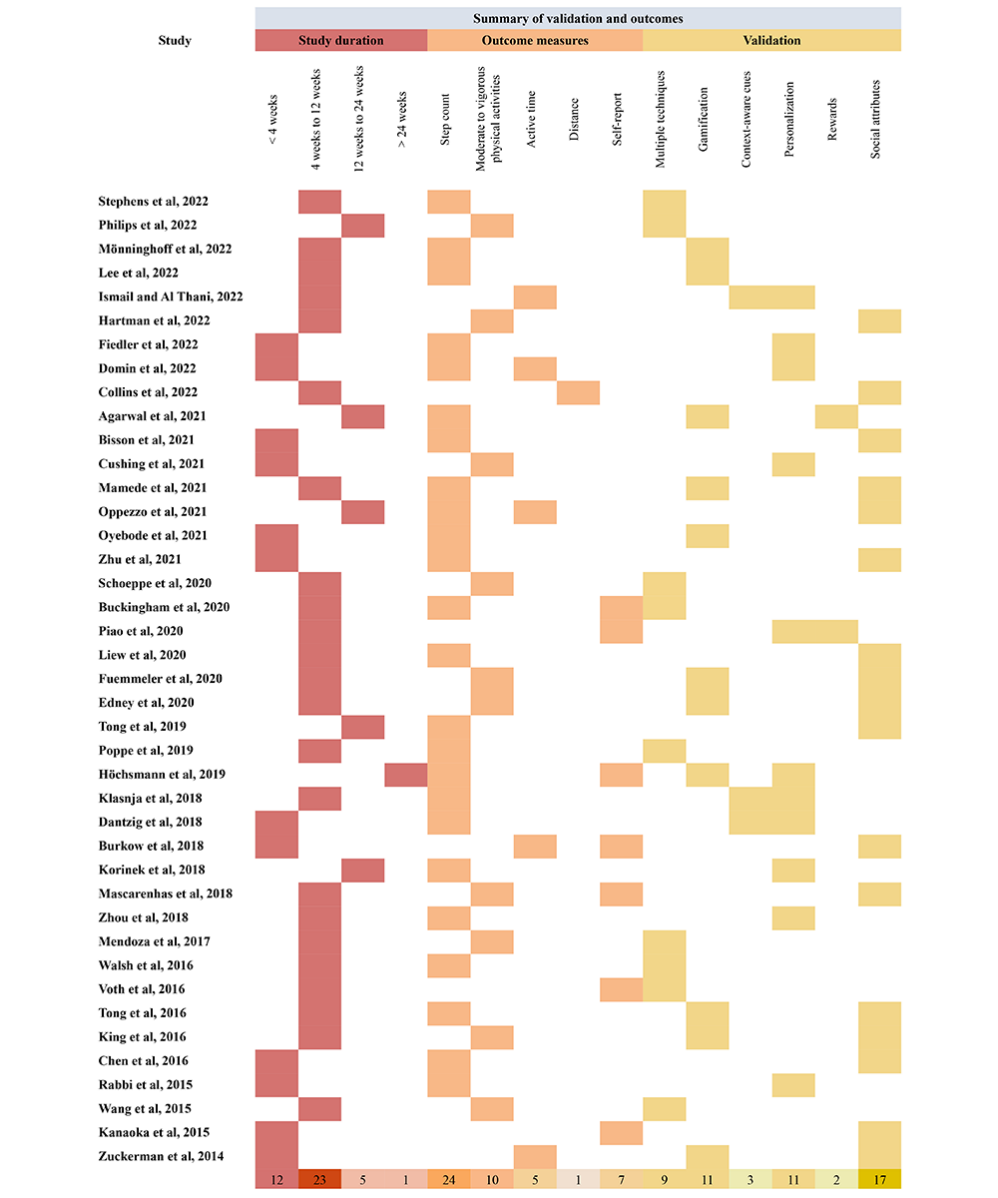
Summary of the study duration, outcome measures, and validation.

**Figure 3:**
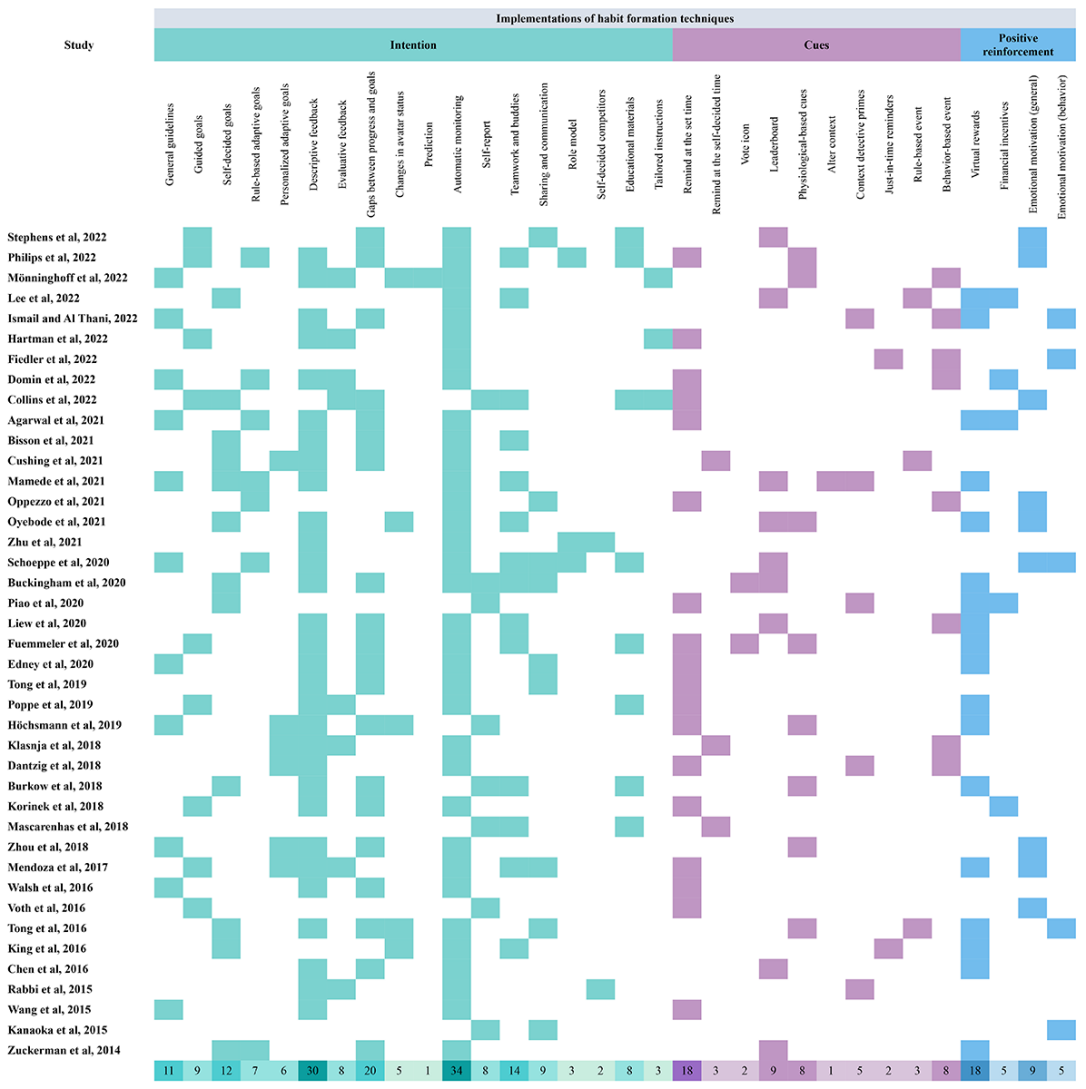
Summary of the design strategies of habit formation techniques.

To visually represent the findings regarding the design objectives and strategies of the selected studies, we present Figures 2 and 3, which are inspired by Bae et al [31]. Figure 2 [32-72] summarizes the study duration, outcome measures, and validation information across 41 studies. Intervention duration ranged from 2 weeks to >2 years. Most articles (36/41, 88%) focused on short-term intervention, with 23 (56%) studies being <3 months and 13 (32%) studies being <1 month. A total of 5 studies focused on medium-term intervention (3-6 months), and only 1 study focused on long-term intervention (>6 months).

Regarding outcome measures, step count was the commonly applied parameter to evaluate the effectiveness of physical activity (26/41, 63%). This was followed by moderate to vigorous physical activities (10/41, 24%). In addition, most studies (34/41, 83%) automatically collected physical activity data from wearables and smartphones, whereas 17% (7/41) of the studies collected subjective data by self-repot.

Themes of validation were analyzed and summarized based on the objectives of the selected studies. A total of 9 articles combined and evaluated multiple BCTs. Besides, social influence was the most evaluated strategy across the selected studies (17/41, 41%), which refers to sharing behavior, teamwork, social support, and social comparison. The gamification theme refers to studies that validate gamified elements (eg, avatars, ranking, levels, and virtual rewards), which was second evaluated strategy in the selected articles (11/41, 27%). Similar to gamification, 11 (27%) articles validated the personalization strategy, including personalized goals, tailored content based on individuals’ performance and goals, and just-in-time reminders. In addition, only 3 (7%) studies investigated the effectiveness of context-aware cues, which refers to strategies that used context data to provide tailored interventions. A total of 2 (5%) articles focused on validating internal and external rewards effectiveness.

### Design Strategies of Habit Formation Techniques

#### Overview

The codebook of habit formation techniques was based on the BCTs [22] and categorized them into 3 labels: implementations to facilitate the intention, cues, and positive reinforcements. The following paragraphs describe each technique. Figure 3 [32-72] presents the summary of the 3 labels of habit formation in the selected studies.

#### Implementations to Facilitate the Intention

These implementations aim to provide strategies that enhance reflective thinking and motivate intention to change behavior, which includes goal-directed behavior, feedback, self-monitoring, social influence, and shaping knowledge.

Goal-directed behavior was coded according to the performers and behavior status. Static goals refer to the fixed objectives that remain unchanged throughout interventions, which include *general guidelines* (eg, walking 10,000 steps a day), *self-decided goals*, and *goals guided by coaches or credible sources*. Most studies (27/41, 66%) adopted static goals across the selected articles (Figure 3). In specific, *self-decided goals* were influenced by information sources, which were divided into self-decided goals based on system-provided plans [32], self-decided goals based on recommendations [33], and allowing to adjust goals during the intervention [34,35]. On the contrary, goals that adapt to changes based on individuals’ behavior change and context information are adaptive goals. It is categorized into 2 types across the selected studies: *rule-based adaptive goals* and *personalized adaptive goals*. A total of 6 articles proposed *rule-based adaptive goals*, which implemented strategies to unlock more complex challenges [36] and goals increased weekly based on set rules [36-40]. In addition, 5 articles applied *personalized adaptive goals,* including human-guided adjustment of goals [41], tailored recommendations of goal setting generated by past behavior and context data [34,42,43], and tailored goals provided by the coaching system [44].

Other strategies also contribute to motivating intentions. First, *feedback and self-monitoring* were the most frequently adopted BCTs across 41 studies. Feedback contains various representations. *Descriptive feedback* refers to information displayed related to individual behavior data that are commonly presented as numerical or diagrams [39,45]. On the contrary, *evaluative feedback* provides comments about individuals’ behavior and goal performances [46,47]. *Gaps between progress and goals* provide explicit information to motivate individuals [48-50]. For instance, Korinek et al [51] used a green bar chart to represent current step counts, and the daily goal was shown as a red line that users directly perceived the gap to the target. Moreover, *changes in avatar status* were commonly applied in gamified systems that linked avatar’s appearances to individual behavior consequences to enhance reflective thinking [46,52,53]. Meanwhile, the *prediction* generated based on analyzing past behavior data provides the consequences of behavior to enhance awareness of maintaining health-enhancing behaviors or reducing health-impairing behaviors [54]. Regarding self-monitoring, most studies *automatically monitor* individual behaviors through smartphones or wearables, whereas some recorded physical activity by *self-report*, such as daily dairy [55-59].

Second, social support and comparison strategies boost behavior change by encouraging and competing with others. *Teamwork and buddies* motivated engagement in physical activity through support from relatives, friends, or team members with consistent goals [59-61]. *Sharing and communication* in the social network enhance behavior change through others’ performances and stories [41,62,63]. Meanwhile, an individual performance develops as a *role model* that influences others’ behavior, especially among family members [37]. In addition, allowing users to choose their competitors (*self-decided competitors*) generated by systems was argued to potentially motivate physical activity levels by comparing with those who performed better than themselves [64].

Finally, information related to shaping knowledge was applied in the selected studies. Health behavior knowledge is shaped through *educational materials* (eg, news) and *tailored instructions* [35-37].

#### Cues Implementations

According to prior studies, cues were classified into 5 categories in the selected articles [20,26]. The most applied strategies were time-based cues (22/41, 54%), such as push notifications at the set time (*remind at the set time*) or *remind at the self-decided time*. The second most applied strategy was social-based cues (10/41, 24%), which refers to cues that stimulate action based on others’ support, the performance of team members, and comparison with others. For instance, *vote icons* (eg, “likes” or “thumbs-up”) were applied to encourage others [65,66]. Displaying others’ information (eg, profiles, status, or progress) was used to boost social comparison for physical activity [64]. Visualization of team members’ performance (eg, *leaderboard*) and team goals were used to boost motivation for walking [39]. However, some instinctive and natural cues, such as physiological-based and context-aware cues, were rarely adopted. *Physiological-based cues* refer to priming by physiological signals and senses that already exist in humans, such as heart rate, smell, and sight. A total of 7 articles applied *physiological-based cues*, especially sight-based strategies. One strategy designed to develop cue-behavior associations involved mapping behavior consequences with virtual avatars. For example, a humanoid avatar was used to represent future health consequences based on current physical activity and nutrition, increasing users’ awareness and motivating behavior change [53]. Another sight-based strategy used nudges to prompt physical activity by *altering context*. Mamede et al [39] used physical table signs in office spaces as motivational nudges to remind and boost physical activity levels and reduce sedentary behavior. In addition, *context detective primes* were argued to provide effective and personalized intervention by integrating context data [43,44,67]. Location, weather, and behavioral event are commonly context detective data that aim to deliver *just-in-time reminder* or recommendation for individuals. Despite context detection potentially prompting physical activity, limited studies focused on investigating the long-term effectiveness of context-aware cues [44,67]. Moreover, 2 articles set specific events by identifying users’ behavior to prompt event-based cues, such as push notifications when users reach their goals or finish their lunch (*rule-based event*) [40,44]. Moreover, event-based cues were tailored solutions based on individuals’ behavior (*behavior-based event*) instead of prompting reminders by setting a specific time.

#### Positive Reinforcement Implementations

Finally, providing internal and external rewards was the commonly adopted strategy for positive reinforcement. *Virtual rewards* (eg, points, badges, and trophies) were the most applied external rewards across the selected studies (16/41, 39%), which commonly used gamified elements to enhance engagement and motivation for physical activity in DBCIs [46,61,68-70]. On the contrary, although *financial incentives* were demonstrated to be more effective as external rewards compared to virtual rewards, their effects were argued to lack long-term sustainability and diminish over time [38,47]. In addition, *emotional motivation* was commonly applied as internal rewards across the selected studies (12/41, 29%). For instance, motivational text messages were provided when users reached the goal by a digital coach (chatbot) [33], whereas some motivational messages that integrated behavior change theories provided general content to boost emotional motivation [35]. Emotional encouragement (eg, verbal thanks, high five, or messages) was used to reinforce behavior change [40,72].

### Research Framework

#### Overview

To better understand and investigate how to leverage the capabilities of digital devices in DBCIs for habit formation, a research framework was developed and defined based on two levels: (1) the target degree, which focuses on the target of techniques; and (2) the technology-mediated interactions, which focus on the characteristics of these technologies in implicitly sensing.

#### Personalization Versus Generalization

DBCIs have gradually developed from providing generalized solutions to personalized interventions for individuals or a category of individuals. Theoretically, personalization represents different conceptualizations based on the research domains. According to the study by Fan and Poole [73], cognitive science defined personalization as “the understanding of the user, the user’s tasks, and the context in which the user accomplishes tasks and goals*.*” In the computer science domain, personalization is “*...*support individualized information inflow and outflow or presenting customers with services that are relevant to their current locations, activities, and surrounding environments*.*”

Hence, in the context of DBCIs, the personalization-generalization dimension refers to the target population of BCTs, specific individuals, and a group of people who share common features or general populations. Meanwhile, personalization means those strategies that collect personal data to potentially impact individual behavior or affect the decision-making of changing behavior.

#### Implicitness Versus Explicitness

Another dimension is technology-mediated interactions. On the one hand, behavior is partly influenced by explicit BCTs, which prompt intention. At the same time, internal cues (eg, emotion, mood, belief, and attitudes) and the surrounding environment subtly influence and guide the association between cues and behavior without consciousness [74], which highlights the importance of the unconscious process and implicit cues. On the other hand, intelligent systems emphasize the ability to sense the environment and analyze the abundance of data for inference and prediction, which also highlights the importance of implicitness during the interaction between humans and intelligent systems.

Ju and Leifer [75] argued that traditional HCI was limited to command-based and graphical interface–based explicit interactions, whereas implicit interactions, defined as “those that occur without the explicit behest or awareness of the user,” are common in day-to-day interactions. Developing on this, Serim and Jacucci [76] described implicit interaction as “...a system response to the user input does not rely on the user having conducted the input to intentionally achieve it*.*” With the development of ubiquitous computing, big data, and AI, implicit interactions increasingly influenced users to interact with the intelligent system because these emerging technologies have the ability to sense the environment that does not rely on intentional input from users. Hence, in this context, implicitness refers to unintentionally taking action, reducing attention during input or output, action without awareness, and executing results without conscious processing [76]. The implicit-explicit interaction dimension refers to whether the design of BCTs requires intentional goals, users’ attention, awareness of input, and conscious processing.

### Framework of the DBCIs for Habit Formation

#### Overview

Therefore, we categorized habit formation techniques based on the following criteria:

- *Generalization*: the technique does not rely on user input and affects the general population.
- *Personalization*: the technique collects personal data that potentially influence the behavior or impact making behavior change decisions.
- *Explicitness*: the technique relates to intentionally achieving or avoiding a specific effect; the input and output processes demand attention and initiative to manipulate, and users are aware of the action outcome during the interaction.
- *Implicitness*: the technique does not rely on the user’s intention to achieve a specific effect; the input or output processes reduce user attention, the action that uses physiological signals without awareness, and execute results without conscious processing during the interaction.

**Figure 4:**
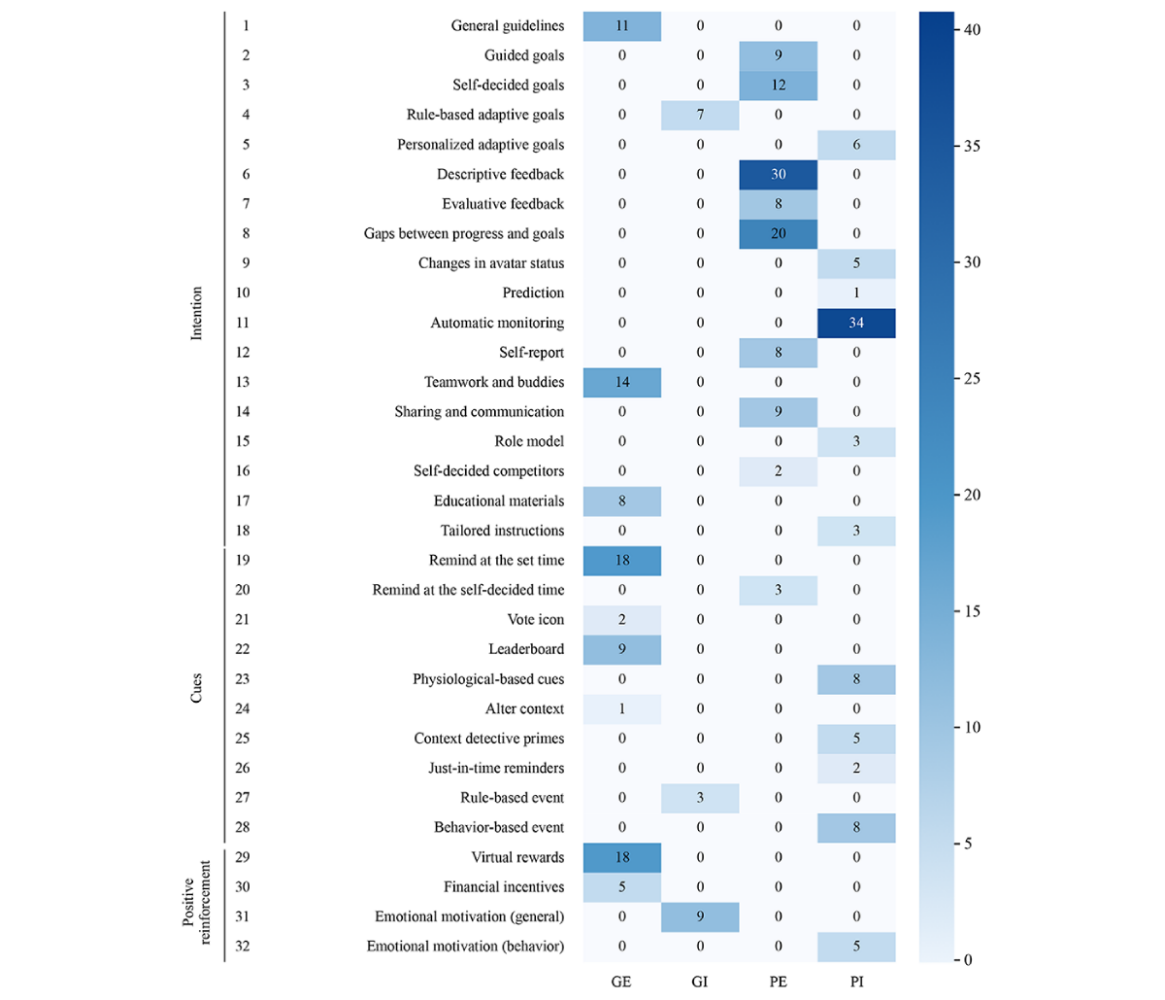
Frequency of habit formation techniques in 4 categories. GE: generalization-explicitness; GI: generalization-implicitness; PE: personalization-explicitness; PI: personalization-implicitness.

Habit formation techniques were positioned along the framework in 4 categories: *generalization-explicitness* (GE), *generalization-implicitness* (GI), *personalization-explicitness* (PE), and *personalization-implicitness* (PI). The frequency of habit formation techniques’ application in each category is summarized in Figure 4. The detailed descriptions of each category are explained in the following subsections and summarized in Table 1.

**Table 1:** Summary of the strengths and weaknesses of each category.

Category	Pros	Cons
Generalization-explicitness	Well-proven effectiveness Broad applicability	It cannot be tailored based on individuals’ situations and preferences The effectiveness of the intervention cannot be maintained in the long term
Personalization-explicitness	Increase implicit belief Focus on background activities	It cannot be adjusted based on individual situations
Generalization-implicitness	Initiative Reflection	It ignores the potential impact from subtle cues that users may not be consciously perceive Inaccurate data The effectiveness of intervention cannot be maintained in the long term
Personalization-implicitness	Unawareness Context detection	Not all health-enhancing behaviors are easily captured Hard to define and identify cues in daily life The effectiveness is questioned Ethic issues

#### GE Category

The characteristics of the GE category are (1) not relying on the user input information, (2) effect works on the general population, and (3) the user intends to achieve goals through applying techniques. A total of 9 techniques are summarized into this category: goals with *general guidelines*, exercise with team members or fitness buddies (*teamwork and buddies*), *educational materials*, push notifications at the set time (*remind at the set time*), *altering context*, *leaderboard* display, voting for others (*vote icon*), and *virtual rewards* or *financial incentives*.

The advantages of GE techniques include well-proven effectiveness and broad applicability. On the one hand, a large number of studies have assessed the effectiveness of goal setting, social support by relatives and friends, notifications, and positive reinforcement for a long time [77-80]. On the other hand, several studies have assessed DBCIs across diverse target groups, demonstrating their broad applicability, including studies involving adults with hypertension [81], individuals with diabetes [82], older adults [83], and children with obesity [84]. Meanwhile, general guidelines serve as credible sources that are widely applied to interventions. Authorities (eg, the World Health Organization, American College of Cardiology, and Centers for Disease Control and Prevention) have published various reports on nonpharmacological intervention approaches to instruct patients with chronic diseases, physicians, and general populations, such as regular physical activity, reduce sodium intake, and weight control [2,85].

However, broad applicability means that the GE techniques cannot be tailored based on individuals’ situations and preferences, while the effectiveness of intervention cannot be maintained in the long term [86]. According to the transtheoretical model, the individual performs different attitudes, skills, and abilities at different stages of the behavior change process, and intervention techniques should be adjusted based on the stage [87]. Therefore, tailoring is considered a vital technique for behavior change interventions [88]. In addition, personal interest and intrinsic motivation are argued to be beneficial to forming habits [89], which also emphasizes the importance of personalization.

#### GI Category

The category of GI contains the following attributes: (1) not relying on the user input information; (2) effect works on the general population; and (3) results in execution do not rely on users’ intention, awareness, or consciousness. Only 3 techniques are included in this category, which are *rule-based adaptive goals*, *rule-based events*, and e*motional motivation (general)*.

The techniques in the GI category contribute to increasing implicit belief. According to Hagger [74], the development of implicit beliefs, attitudes, and affective reactions potentially enhances habit formation. Hence, techniques such as graded tasks are applied to motivate positive belief. Graded tasks refer to increasingly difficult but achievable small tasks that are divided by long-term goals [22]. Despite the difficulty of the tasks gradually increasing, a stronger belief (eg, “I can do it!”) is formed when the user achieves the tasks. Meanwhile, some studies provide general motivational messages to enhance the development of belief by applying the Health Belief Model [90]. However, high cognition and awareness are required to develop and reinforce the belief, which is challenged by individual capabilities. Hence, the GI techniques assist in reducing the requirements of user capabilities by focusing on background activities. Background activities are defined as “interactions that elude the user’s attention” [75]. The rule-based techniques are established to achieve behavior changes that do not rely on the user’s intentionality or initiative. In other words, the rule-based techniques are developed according to the effect, which is not limited to individual cognitive processes. For instance, the next level of challenges was unlocked after achieving the event (eg, accomplishing previous levels’ tasks) in gamified interventions [36]. Goals were adjusted weekly, increasing in difficulty by 10% according to predetermined rules [36-40].

However, rule-based techniques still cannot be adjusted based on individual situations. Although the GI techniques try to provide event-based influences, the individual behavior change process is not static and consistent, impacting the context. Meanwhile, the development of belief is also a highly individual process. Hence, generalized solutions have limited effects.

#### PE Category

The category of PE contains the following capabilities: (1) tracking personal data, (2) effect generated according to individual input, (3) techniques assist users in achieving their goals, and (4) outcomes aim to enhance reflection. A total of 9 techniques belong to the PE category, including *guided goals*, *self-decided goals*, *descriptive feedback*, *evaluative feedback*, *gaps between progress and goals*, *self-report*, *sharing and communication*, *self-decided competitors*, and push notifications at the self-decided time (*remind at the self-decided time*).

Initiative and reflection are strengths of the PE techniques. On the one hand, the user initiates behavior with high intentionality. Users actively make decisions, and effects are decided by what users intend to achieve. Hence, the PE techniques are applied to tailor for individuals. For example, self-decided goals provide autonomy that users can set or adjust the detailed plans according to their current situation, which is perceived as more persuasive by users [91]. On the other hand, personal information intends to help users’ self-reflection to take action [92]. Explicit feedback is generated based on individual input that draws the individual’s attention, which aims to reflect individual historical behavior to facilitate self-reflection.

However, the PE techniques rely too much on individual initiative and intentionality, which ignores the potential influence of subtle cues that users may not consciously perceive. Self-report data are argued to be inaccurate and influenced by individual cognitive bias [20]. Moreover, displaying individual information is argued to be useless for individual and cannot maintain long-term effectiveness [11]. Barriers such as inaccurate data, unclear feedback, and insufficient explanation are reasons for abandoned monitoring [93].

#### PI Category

The characteristics of the PI category are (1) tracking personal data; (2) effect generated according to individual input; (3) action without awareness; and (4) results in execution do not rely on users’ intention, awareness, or consciousness. A total of 11 techniques are summarized in the PI category, which includes *personalized adaptive goals*, *changes in avatar status*, *prediction*, *automatic monitoring*, *role model*, *tailored instructions*, *behavior-based events*, *just-in-time reminders*, *context detective primes*, *physiological-based cues*, and *emotional motivation (behavior)*.

Unawareness and context detection are advantages of the PI category. Unawareness occurs during the whole process of DBCIs. First, users may not be aware of what health data will be collected. Second, user behavior may be constantly monitored without awareness through wearables, cameras, or other IoT devices. Third, collected information not only includes individual behavior data but also contains context data without intentionality. Multiple dimensions of data are integrated to identify problems and patterns, which are prepared for generating meaningful effects for individuals. Then, users may not be aware of using physiological signals (eg, heart rate, gaze, and smell) when they take action. Finally, effects may not rely on the user’s cognitive process. Since the development of IoT, big data, and AI, techniques in the PI category could assist in achieving the above unawareness process. For instance, various sensors are embedded in IoT devices that support constant monitoring of individual behavior, physiological signals, and context data. A large amount of data contributes to recognizing patterns with similar conditions and real-time analysis of mood and context. In addition, the PI techniques are good at identifying implicit and subtle context cues in daily life, which users easily ignore. Context detection is argued to play a vital role in habit formation [20,94]. Ideally, habitual behaviors are repeatedly triggered by suitable context cues that can be detected stably and constantly in daily life [15]. Hence, identifying suitable cues for individuals could enhance the development of cues-behavior association to respond at the right time.

However, PI techniques have faced various challenges since adopting emerging technologies. First, not all health-enhancing behaviors are easily captured. For instance, accelerometers in smartphones or wearables could automatically track physical activity, such as steps or sedentary behavior [70,72]. On the contrary, information on eating behavior, such as calorie intake, is more difficult to capture [56]. Second, it is hard to define and identify cues in daily life [20,95]. Location and time cues are easy to track, so existing DBCI studies focus on time-based cues. Other types of cues (eg, mood or physiology signals) are rare to explore in terms of how they develop a connection with behaviors. Third, although existing DBCIs attempt to provide just-in-time reminders to impact user behavior, their effectiveness has been questioned [67,94]. Finally, ethical issues must be considered because of the implicitness and unawareness of PI techniques. For instance, privacy and trust issues are worrisome challenges because the reasoning process of intelligent systems is obscured, and the status of personal health data is untraced [96,97].

## Discussion

### Principal Findings

**Figure 5:**
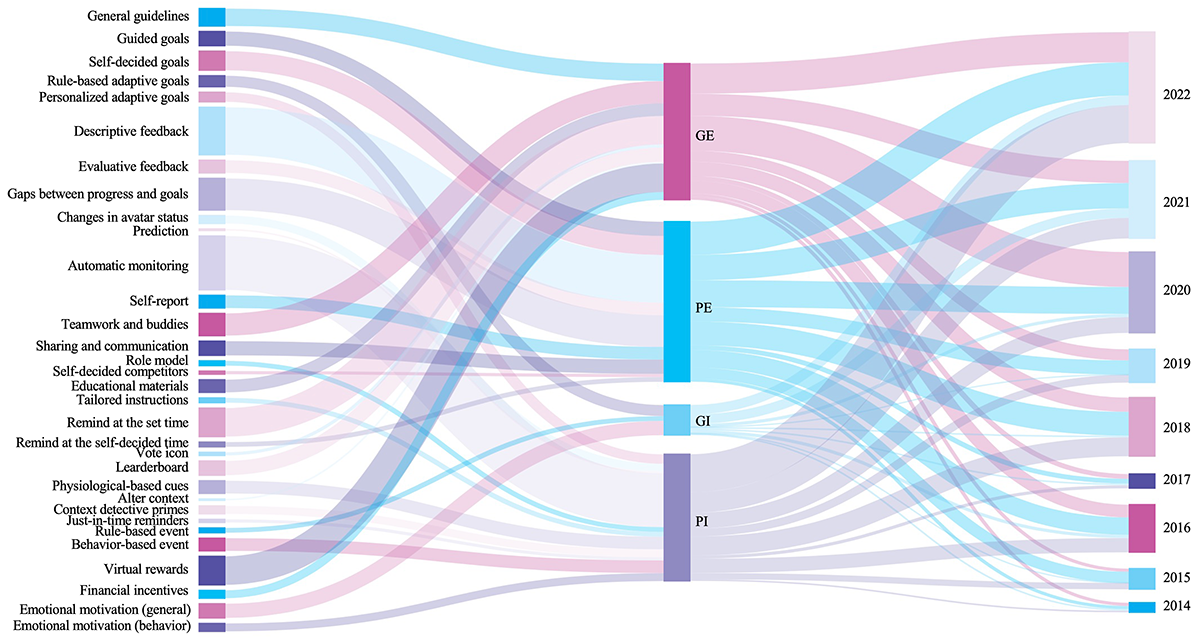
Relationship between habit formation techniques (left), categories (middle), and years (right). The bar widths indicate frequencies of each item. GE: generalization-explicitness; GI: generalization-implicitness; PE: personalization-explicitness; PI: personalization-implicitness.

Habit formation is an iterative and cyclical long-term process. To gain a deeper understanding of how to adopt habit formation techniques in designing DBCIs, this study examines techniques from the design perspective of user target–mediated and technology-mediated interactions. The identified habit formation techniques are categorized into 4 main dimensions: *generalization*, *personalization*, *explicitness*, and *implicitness*. As shown in Figure 5, techniques of the GE and PE category have been widely implemented in DBCI solutions, such as *descriptive feedback*, *teamwork and buddies*, *remind at the set time*, and *virtual rewards*. These techniques demonstrate more applicability that could be adopted by a wide range of populations. Meanwhile, GE and PE techniques partly replace the role of traditional interventions, which reduces the burden of knowledge requirement and is beneficial to the implication of self-management in a low-cost manner [98]. Furthermore, there has been a growing use of GI and PI technologies after 2016, particularly automatic monitoring techniques. The development of wearable devices has allowed for the collection of an increasing amount of behavioral and physiological data. Studies focus on using emerging technologies (IoT, big data, and AI) to detect subtle cues and reduce user effort and attention [7,9,26], which indicates the trend of using techniques of PI category. On the basis of the reviewed literature, we summarize the existing habit formation techniques into the following categories: habit formation process, challenges, and potential solutions (Table 2). The following subsections provide detailed discussion of each category.

**Table 2:** Summary of techniques in the habit formation process, challenges, and potential solutions.

Habit formation	Challenges	Potential solutions
Intention and action control	Most of the explicit techniques still rely on users’ attention and frequently interact with the device, which demands high initiative	Applying implicit techniques to reduce the demand for users’ attention and motivation
	Identifying and defining unexpected events	Capturing contextualized data as historical records, which serve as a reference for identifying deviations from the norm or unusual data, enabling the inference of unexpected events
	Providing coping planning to deal with unexpected events	To achieve predictions and tailored recommendations, it is necessary to capture not only common data such as weather, location, time, and activity but also users’ physiological data, emotions, social relationships, and other relevant data
Developing association between cues and behaviors	Low user response to pushing notification	Just-in-time adaptive interventions
	The cue-behavior association is a fast, automatic, and subtle process	Establishing a stable repetition of implicit cues in the context, such as incorporating other types of cues and leveraging preexisting associations
Providing personalized response	Useful and meaningful data to enhance motivations and intentions to change behavior	Identifying the specific data points that hold significance for users and effectively predict users’ preferences is crucial for facilitating meaningful self-reflection and behavior change
Others	Data privacy	Developing effective techniques for tailored interventions and recommendations based on individuals’ preferences Addressing the need to present transparent information to raise awareness of data privacy
	Perceived inaccuracy	Improving sensor technology and processing algorithms to calibrate the accuracy of the tracking device Transparency design methods that explain device abilities and limitations could enhance user trust and calibrate user expectations
	Usability and comfort issues	Ideally, users should feel physically comfortable wearing and intuitive to use the device

### Forming Intention and Action Control

To form habits, people make decisions about goal achievement first. Subsequently, intentions are transferred into action through action planning [15,16,89]. Existing habit formation techniques, especially explicitness techniques (GE and PE), have been well-supported goal setting and action planning, such as *guided goals* and *self-decided goals*. Most of these explicit techniques still rely on users’ attention and frequently interact with the device, which demands high initiative. Studies have applied implicit techniques to reduce the demand for users’ attention and motivation. For instance, *rule-based adaptive goals* aim to automatically provide graded tasks to users without their awareness, which is based on time, progress, or users’ performance.

However, the process of transferring intention to actions is influenced by unexpected events [99]. Unexpected events disrupt users’ established routines, leading to an inability to consistently repeat behaviors in a stable environment. Few empirical studies have explored how to create and execute alternative goals and plans to deal with these unexpected events. One of the challenges is the identification and definition of unexpected events. Existing studies focus on capturing common contextualized data (eg, weather, location, temporal factors, and activity data) as historical records [99,100]. These records serve as a reference for identifying deviations from the norm or unusual data, enabling the inference of unexpected events. However, the influences of other implicit factors are disregarded in current studies, such as social activities and the chain reactions caused by unfinished tasks.

Another challenge is providing coping planning to deal with unexpected events. The concept of coping planning is proposed as a self-regulation method, which refers to identifying problems and obstacles that may impact planning and developing alternative plans to overcome these challenges [101]. Ideally, intelligent systems predict the point of unexpected events based on learning individual behavior and preferences. Meanwhile, multiple alternative plans could be suggested according to users’ mental status and context information, such as suggesting alternative activity times or activity types that are suitable for the current context. To achieve predictions and tailored recommendations, it is necessary to capture not only common data such as weather, location, time, and activity but also users’ physiological data, emotions, social relationships, and other relevant data.

### Developing Cue-Behavior Associations

Regarding developing the association between cues and behavior, most of the existing studies are limited to static and fixed time-based cues (*remind at the set time*). This GE technique cannot adapt to individual situations to maintain long-term behavior change. Moreover, pushing notifications is facing the challenge of low user response [67]. Users might notice the notifications but may not respond; in other words, users may not act on reminders immediately. Integrating mobile sensing technology to deliver notifications at the right time based on personalized needs is argued to be one of the solutions. Prior studies have used the just-in-time adaptive interventions for various health behavior changes, such as weight management [71], dietary intake [56], and physical activity [67,102]. However, just-in-time adaptive interventions rely on factors such as type, time, and intensity, which ignores other context cues essential in developing cue-behavior associations for habit formation [20].

In addition, the cue-behavior association is a fast, automatic, and subtle process. Instead, *remind at the set time* is an explicit interaction that demands users’ attention and initiative to respond, which is a slow and conscious goal-directed process. The development of association should pay more attention to instinctive signals and implicit cues [20,26]. It is crucial to explore potential solutions for establishing a stable repetition of implicit cues in the context. This can be achieved by incorporating other types of cues (eg, physiological signals, auditory responses, and visual cues) and leveraging preexisting associations (eg, red color associates with stop). By implementing these strategies, the effectiveness of implicit cues could be enhanced in influencing and guiding individuals’ behaviors.

### Personalized Response

Furthermore, personalized response plays a major role in DBCIs, which can stimulate users’ self-reflection and motivation for changing behavior. As shown in Figure 4, studies have adopted various techniques to satisfy individual requirements, such as *descriptive feedback*, *gaps between progress and goals*, *just-in-time reminders*, and *role model*. However, most of the responses only present data through various types of visualized graphics [36-38,103,104]. For instance, similar types of user data are applied to facilitate individual reflection on their behavior [104]. Peaks and valleys are used to demonstrate the extreme value that may explain the reasons for their behavior. Visualizing the relationship between users’ activity trajectory and the target progress could help enhance user awareness [105].

Users need to comprehend the meaning of these data and engage in self-reflection regarding their own actions. A major challenge lies in determining what data are useful and meaningful to individuals to enhance motivations and intentions to change behavior. Identifying the specific data points that hold significance for users and effectively predict users’ preferences is crucial for facilitating meaningful self-reflection and behavior change. For instance, Zhu et al [64] proposed a model to personally recommend virtual fitness buddy profiles to users by capturing daily step data, which aimed to examine whether individuals reacted positively when they compared with those who performed better than themselves. However, their findings found the personalization paradox problem, which means that personalization models may inaccurately predict users’ preferences because the model is developed based on daily steps. Various contexts and implicit information about individuals cannot be captured. Hence, individuals perceive the uselessness and inaccuracy of proposed solutions.

### Other Challenges

Several research challenges should be taken into account in the future design of DBCIs. First, constant monitoring of the human body and surroundings is a critical feature of DBCIs. It raises concerns of data privacy violations, such as collecting data without users’ permission or unauthorized trading personal data with third parties. To address these privacy issues, regulations have been implemented to safeguard data privacy. In addition, studies focus on proposing transparency design principles to enhance users’ awareness of data privacy [97]. Future studies should not only focus on developing effective techniques for tailored interventions and recommendations based on individuals’ preferences but also address the need to present transparent information to raise awareness of data privacy. Second, the perception of inaccuracy in tracking devices is another challenge [11]. Users may question the reliability of the data collected by these devices, especially when the feedback from the tracking does not align with their subjective perceptions and expectations. Considering this discrepancy may lead to a loss of trust and confidence in the DBCIs, future studies should improve sensor technology and processing algorithms to calibrate the accuracy of the tracking device. Meanwhile, transparency design methods that explain device abilities and limitations could enhance user trust and calibrate user expectations. Finally, to achieve long-term behavior change to form habits, usability and comfort issues are important considerations to constantly adopting smart devices [11,14]. Ideally, users should find the device comfortable to wear and intuitive to use. Addressing these concerns will contribute to long-term engagement with these smart devices and enhance the effectiveness of DBCIs.

### Limitations

Although we used the standard systematic review method to identify relevant articles, there are still limitations of the study, such as scope of the database and availability of data perspectives. As we aim to review the design implementations of DBCIs, it is possible to select the target databases from the HCI field (ACM Digital Library) and focus on the design process and techniques of interventions. Another limitation is that some articles might be missed because they did not include the validation processes. Meanwhile, some selected papers only had quantitative data in validation processes or conducted multiple techniques, which makes it hard to identify the effectiveness of implementations of DBCIs. Hence, future studies could also include the efficacy of a single technique or the combination of DBCIs in consideration.

### Conclusions

This study presents a systematic review aimed at identifying the design strategies of habit formation techniques in existing DBCI studies for physical activity. The findings revealed the existence of gaps between behavior change theories, exploration of technology capabilities, and design strategies for long-term adoptions of DBCIs. By examining design approaches in current DBCIs for physical activity with a focus on design targets and technology capabilities, a conceptual framework was proposed to investigate the prospects for design strategies of habit formation.

The findings indicate that the most applied BCTs include self-monitoring of behavior, goal setting, and prompts and cues. Mapping to the design strategies of habit formation techniques in the conceptual framework, most studies used *automatic monitoring* and *descriptive feedback* techniques, which commonly apply various sensors in devices. Moreover, existing research emphasizes the use of explicit interaction in design strategies. For instance, *general guidelines* and *self-decided goals* were widely used to facilitate behavior intention. Time-based cues (*remind at the set time*) and *virtual rewards* were commonly applied cues and positive reinforcement. Meanwhile, there is a clear trend highlighting the significance of personalized habit formation techniques in DBCIs’ design strategies. This not only necessitates exploring technological characteristics but also addresses the specific needs of individual users. However, implicit interaction design strategies of habit formation techniques are lacking in the reviewed studies. Potential challenges include difficulty in capturing all health-enhancing behaviors, defining and identifying various cues in daily life, and ethical considerations regarding the unawareness of automatic tracking behavior.

The outcome of the conceptual framework and potential solutions can serve as guidelines for further DBCIs’ design strategies of habit formation. Future studies are suggested to investigate the implementations to facilitate the development of cue-behavior associations, especially those implicit interactions that adopt emerging technologies’ features.

## Appendix

Multimedia Appendix 1 PRISMA checklist.

## Abbreviations

AI: artificial intelligence
BCT: behavior change technique
DBCI: digital behavior change intervention
GE: generalization-explicitness
GI: generalization-implicitness
HCI: human-computer interaction
IoT: Internet of Things
PE: personalization-explicitness
PI: personalization-implicitness
PRISMA: Preferred Reporting Item for Systematic Reviews and Meta-Analyses
RQ: research question
